# De novo transcriptome sequencing in *Monsonia burkeana* revealed putative genes for key metabolic pathways involved in tea quality and medicinal value

**DOI:** 10.1007/s13205-016-0563-y

**Published:** 2016-11-19

**Authors:** Adugna A. Woldesemayat, Khayalethu Ntushelo, David M. Modise

**Affiliations:** Department of Agriculture and Animal Health, College of Agriculture and Environmental Sciences, University of South Africa, UNISA Science Campus, Corner of Christiaan De Wet Road and Pioneer Avenue, Florida 1710, Johannesburg, South Africa

**Keywords:** De novo assembly, *Monsonia burkeana*, Functional annotation, Illumina sequencing, Metabolic pathways, Transcriptome

## Abstract

Monsonia (*Monsonia burkeana* Planch. ex Harv) is one of the most valuable tea and traditional medicinal plants used in Southern Africa. In spite of this, there is no sequence information regarding this plant in literature. To provide understanding of the naturally occurring tea and drug-specific products and the key pathways for the biosynthesis of these molecules, we sequenced the leaf transcriptome using Illumina MiSeq platform and generated 2,590,652 paired-end reads that were assembled de novo into 45,450 high-quality transcripts. Annotation of these transcripts revealed best hits for homology to discover more than 17,800 functional genes and conserved domains. A total of 93 KEGG pathways and associated genes encoded by more than 90% of the coding transcripts are responsible for the biosynthesis of these life-saving metabolites. We validated and enriched the genes by GO annotation and linked this to enzyme-powered pathways through interactive network map. Caffeine metabolism, flavonoid, phenylpropanoid and terpenoids biosynthesis and xenobiotics degradation were typical in tea quality and drug therapy. The relatedness of more than 80 gene families encoding key enzymes was shown using unrooted phylogenetic tree. In conclusion, the *M. burkeana* leaf transcriptome gives insight into tea and drug-specific products, therefore representing basis in further investigation of the plant.

**Electronic supplementary material:**

The online version of this article (doi:10.1007/s13205-016-0563-y) contains supplementary material, which is available to authorized users.

## Introduction

The emergence of next generation sequencing (NGS) has provided immense practical advantages for comprehensive analysis of sequences at the genome and transcriptome level, through widely accessible massively parallel short-reads sequencing platforms. Today, we are able to witness these rapidly evolving novel technologies that require automated protocols for generating sequencing libraries with effective and different approaches to genomic sequence analysis and to experimental designs. Thus, DNA sequencing and genome assembly, and RNA sequencing and transcriptome assembly remain to be ongoing projects as a corner stone for comprehensive sequence-based analysis that enables addressing of novel questions in most areas of biological and agricultural research.

Sequencing the transcriptome is a fast, cost-effective and viable alternative to sequencing the genome and provides a snapshot of expressed genes. Recent advances in techniques used in studies on gene expression and genome mapping evolved to converge on RNA sequencing as a comprehensive and efficient way to measure transcriptome composition, obtain RNA expression patterns, and discover new genes and gene features and accurate transcriptome quantification for single cells (Morozova et al. 2009; Wu et al. 2014). Identification of plant products that have values for human health using the transcriptome assembly is currently widely applicable both in the reference-guided and de novo fragments particularly in non-model plant species whose genome sequence is as yet unavailable or incomplete. In the current study, we embarked on de novo sequence assembly and profiling of a partial transcriptome for the tea plant, Monsonia (*Monsonia burkeana* Planch. ex Harv), Geraniaceae family. *Monsonia burkeana* is one of the members of the genus Monsonia (Touloumenidou et al. 2007) whose genomic information in the public domain is very limited; however, is predominately used as traditional medicine; and has increasingly received attention in terms of harvestable biochemical constituent properties (Tshivhandekano et al. 2014). This quality presents a case for its commercial production particularly for tea beverages that are well known for their abundant secondary metabolites such as polyphenols, theanine, and volatile oils (Rogers et al. 2008).

Naturally occurring compounds are increasingly used as dietary and beverage antioxidants due to their health and environmentally friendly features (Balasundram et al. 2006). This suggests that *M. burkeana* has a potential to provide a dietary essential nutrient elements required for human health benefits. The usefulness of special tea as well as the increasing demand for healthy tea beverages warrant its commercial production. This crop with such a potentially significant economic impact has not yet to our knowledge had its genetic profile investigated. Previous studies have developed transcriptomic resources and established information database in other medicinally important plants using NGS to identify and discover genes that take part in the biosynthesis of secondary metabolite (Shi et al. 2011; Gupta et al. 2013).

Owing to the lack of currently available genomic information regarding *M. burkeana*, it was incumbent to generate an initial dataset de novo starting with RNA sequencing just from one but most informative tissue in view of the practical application of the herbal traditional medicine of this plant. Furthermore, based on the primary interest to develop a strategy to discover the major genes involved in the biosynthesis of secondary metabolites that may contribute to its commercial attributes, it was imperative to initially asses the viability and feasibility of the study before embarking on a comprehensive in-depth transcriptome analysis. However, we believe that further sequencing that includes additional tissues is required to comprehensively cover the transcriptome of this plant. This study, therefore, used the application of NGS towards characterization and profiling of de novo transcriptome sequencing events occurring in leaf tissue using RNA sequencing technology to address the foregoing concerns in *M. burkeana*. The Illumina MiSeq platform that generates RNA-seq short read sequences to add new data, was utilized. It is envisaged that this dataset will add basic knowledge by providing the first-hand transcript sequences of the leaf tissue and promote the basic understanding of the molecular mechanisms of cellular metabolism for future genetic and genomic studies on *M. burkeana* specifically in relation with secondary metabolites.

## Materials and methods

### Plant materials

**Fig. 1:**
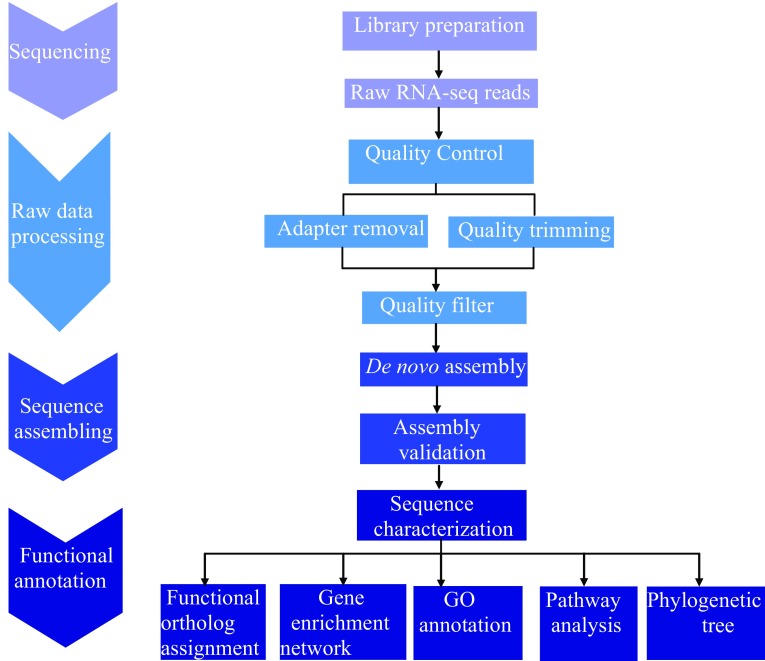
A workflow for de novo sequence assembly and annotation of *M. burkeana* leaf transcriptome. The color of the arrows correspond to the color of a particular process in the work flow. This work flow consists of four major processes. (1) Sequencing reads, include procedures for experimental design, sample and library preparation and generation of Illumina RNA-seq paired-end fastq data. (2) Raw data processing (pre-processing), include filtering the low-quality reads, removing adaptor sequences and errors to optimize and improve the read quality and to facilitate the work of the assembler. (3) Standard analysis (assembly), de novo reconstruction of the quality filtered reads, while maintaining the quality of assembly with the N50, mapping of reads for internal validation and validation of the coding regions using Transdicoder. (4) Special analysis (post-assembly analysis), implement various downstream analysis to functionally annotate the assembled transcriptome

Leaf tissue samples of the *M. burkeana* plants were collected from Chuenespoort (Lat: 24.16188S; Lon:29.48445E), Limpopo Province, South Africa. Frozen tissues were ground to a fine powder using liquid nitrogen so as to preserve RNA integrity. A total RNA samples were extracted separately using the Qiagen RNeasy^®^ Mini Kit. After depleting rRNA from the sample, mRNA samples were quantified using fluorimetry (model 6285, Jenway). Illumina sequencing libraries were prepared using the ScriptSeq mRNA-Seq Library Preparation Kit (Illumina) and then sequenced on the Illumina MiSeq platform at the Agricultural Research Council (ARC) Biotechnology Platform, Pretoria. A total of 2,590,652 paired-end RNA-seq reads were generated and then subjected to the quality assessment protocol. Figure 1 shows the pipeline of the complete process including de novo assembly and annotation of the *M. burkeana* leaf transcriptome.

### Reads quality control (QC) and trimming

FASTX-Toolkit (Gordon and Hannon 2010) and FastQC package (Andrews 2010) were used for raw read quality filtering. FastQC was used to check the quality of the reads before and after processing. FASTX-Toolkit was used to optimize the read quality by removing barcode tags and adaptor sequences using fastx clipper and trim the sequence reads based on the minimum read quality score and on the read length. However, before setting the quality score for filtering the reads above the threshold level, we assessed the outcome of quality scores under various thresholds both in terms of the quality and length of the reads. This multi-step assessment of read quality was undertaken for the quality scores ranging from “Q22 to Q40” at every 3 quality score increment and for the read length ranging from “31 to 100 nucleotides” at an interval of every 10 bases for shorter reads (<80 bases) and at every 5 bases interval for relatively longer reads (>80 bases). This was implemented by flagging quality scores with −q 22, −q 25, −q 28, −q 31, −q 34, −q 37 and −q 40 and length values with −l 50, −l 60, −l 70, −l 80, −l 85, −l 90 and −l 95 where −q representing minimum quality threshold and −l minimum length of the read to be retained. This repeated rounds of quality trimming with variations in quality score and minimum length, was targeted to optimize the read quality for improved de novo assembly. Quality filtering as a final step was carried out based on the best performing threshold using a fastq_quality_filter, a tool incorporated in a Fastx-Toolkit. The processed reads were then evaluated for final quality using FastQC and then compared with the initial assessment results. The reads whose partner were removed based on the minimum threshold quality score and length, were considered as singletons.

### De novo assembly

Velvet v.1.2.10 (Zerbino and Birney 2008) and Oases v.0.1.8 (Schulz et al. 2012) were used to assemble the RNA-seq reads after quality assessment because these tools are shown to be effective in quality transcript assemblies that are based on short-read datasets (Garg et al. 2011; Ness et al. 2011). To run Velveth, the parameters that define the read types and *k*-mer length were sorted to establish the hash-table based on the available *k*-meric sub-sequences that was created in RNA-seq dataset building sequence information for the next step, velvetg (Grabherr et al. 2011). Velvetg was run separately on each directory created by velveth to build De Bruijn graphs (Zerbino and Birney 2008) from the *k*-mers having different length obtained by velveth. By running simplification and error correction over the graph, velvetg extracts with other numerous files the contigs longer than twice the *k*-mer length used in velveth (Grabherr et al. 2011). Oases was used to build de novo transcriptome as it enables to use the advantage of combining the use of multiple *k*-mers to assemble de Bruijn graph by handling alternative splicing variants and by using RNA-seq short read data based dynamic error correction for implementing a robust method to predict full length transcripts (Schulz et al. 2012). A single *k*-mer length is not sufficient to provide the best assembly for a transcriptome in line with the variability of transcriptome coverage related to the extent of gene expression level (Surget-Groba and Montoya-Burgos 2010). For this reason, we ran Velvet and Oases using the *k*-mer range (*k* = 19, 23, 27, 31, 35) to employ additive multiple *k*-mer assembly method.

### Validation and annotation of assembly

We employed various strategies to validate the de novo assembled leaf transcriptome. First, to identify the transcripts that were misassembled, we used an internal validation method that represents a strategy in which reads are mapped back to their own transcript assemblies as a reference to determine miss-assembled candidates using Tophat2 (Tophat version 2.0.14.Linux_x86_64) (Kim et al. 2013) which also uses the short read aligner Bowtie (Langmead and Salzberg 2012). On top of its high speed, TopHat2 identifies potential splice sites for introns so as to accurately align reads of differing lengths to the specific reference transcripts allowing insertion or deletion of bases with high sensitivity unlike most other RNA-seq aligners (Kim et al. 2013). Second, we identified the Open Reading Frames (ORFs) that contain at least 300 nucleotides (100 amino acids long) using the TRANSDECODER Version 2.0.1 (Haas et al. 2013).

The best transcripts selected by the Transdecoder as the valid coding sequences were used in the sequence similarity search against non-redundant (NR) NCBI, UNIPROT and PFAM protein databases. The best hits with known proteins were identified using Basic Local Alignment Search Tool (BLASTP) (Altschul et al. 1990) and Pfamscan (Finn et al. 2016) and were filtered based on the bit score, cut-off e-value 1e-10 and the sequence identity more than 80%. Target protein match were identified in terms of annotation status and the most closely related organism were also identified. PFAM analysis was undertaken to look for transcript sequences assigned to known protein families or domains. Peptides belonging to different and to a specific functional classes were sorted by identifying clan annotation.

### Functional annotation and gene ontology (GO) analysis

The GO analysis was performed to assign function to each validated transcript assembly based on the annotation option using Blast2GO standalone version 3.2 (Conesa et al. 2005). This functional annotation categorized the candidate coding transcripts under all GO categories that include Biological Processes (BP), Cellular Components (CC) and Molecular Functions (MF). The GO functional enrichment analysis was set to *e*-value less than 1e-6 and default threshold values for GO annotation, GO-weight and high scoring alignment pairs (HSPs) coverage. Blast2GO employed a BlastP program, to search for matching protein sequences against NR NCBI database. All annotations are associated to an evidence code which provides information about the quality of this functional assignment (Camon et al. 2004). While the GO IDs of all the transcripts from leaf tissue were retrieved from the Blast2GO annotation database, each annotated transcript sequence was associated to one or more GO-terms but in the same or different GO category (Ashburner et al. 2000). The occurrences of GO terms assigned to each transcript were compared to the occurrences of the background set of GO-annotated transcripts in the entire database using the hypergeometric distribution.

### Pathway analysis

Blast2GO software that incorporates locally installed Blast2GO database using MYSQL DB was used to analyze the de novo assembled validated transcript sequences associated with metabolic pathways. Typical pathways that involve the major genes were identified from the Kyoto Encyclopedia of Genes and Genomes (KEGG) database (Ogata et al. 1999) using mapping option of the Blast2GO software. While Blast2GO provides multiple options of analysis features in parallel such as Enzyme Commission (EC), the KEGG pathway maps, InterPro and the GO annotation, the default parameters were used for blasting and mapping of the sequences to the GO annotations and for identifying the key pathways. The assignment of the EC numbers to transcript sequences was based on the similarity search against NR protein databases using BlastX search algorithm (Altschul et al. 1990).

### Gene enrichment network

The gene enrichment network map was constructed using Cytoscape version 3.3.0 (Shannon et al. 2003) for the genes associated with enriched GO-terms. The GO-accessions were retrieved from the Blast2GO annotation output and submitted to AgriGO (Du et al. 2010) using customized annotation for *M. burkeana* leaf coding transcripts. This was shown as an interactive biological networks based on the coding transcript sequences enriched for GO-terms related to the molecular functions of the GO categories.

### Phylogeny

Phylogenetics is not only important to understand the current evolutionary status of genes, genomes and species, but also useful to predict their future evolutionary fate (Timme et al. 2012). We classified and show the relatedness of the gene families involved in the biosynthetic pathways associated with primary and secondary products in *M. burkeana* leaf tissue using interactive Tree of Life (iTOL) (Letunic and Bork 2011). This was finally represented by the unrooted cladogram phylogenetic tree of life to show the classification and relationship of the gene families that encode the key enzymes involved in the major metabolic pathways.

## Results

### Quality assessment

Sequencing of the *M. burkeana* leaf tissue mRNA on the Illumina MiSeq platform provided a total of approximately 2.6 million RNA-seq paired-end (300 × 300) reads. Given Q20 as the overall per base quality score (Q%) for the raw reads with mean read error rate less than 1%, the percentage of error-free reads was 91.2%, whereas the percentage of ambiguous N bases (N%) was 0.07%. The first concern in sequencing reads analysis for annotating new sequences is about the quality score. The low-quality reads affect the quality of de novo sequencing and downstream analysis due to its increased misassembly rate and the unrealistically represented sequencing coverage based on mere presence of the redundant reads (Zhang et al. 2011), thus undergone quality filtering procedure. The overall GC content for the trimmed and quality filtered reads was 45.6% with the 20% Phred quality score increased to a base call accuracy of 99.98% (Q37 percentage). Based on this minimum quality (−q 37) and the minimum length (−l 50) for all the samples, a total of 1,656,822 quality reads was resulted. Table 1 gives a brief description of the number of reads handled before and after quality control.

**Table 1:** Summarized statistics of the reads before and after quality control

Reads	Before QC	After QC	Sorted	Trimmed	Singletons
Forward	1,295,326	1,075,915	473,006	219,411	602,909
Reverse	1,295,326	580,907	473,006	714,419	107,901
Total	2,590,652	1,656,822	946,012	933,830	710,810

### De novo assembly

**Fig. 2:**
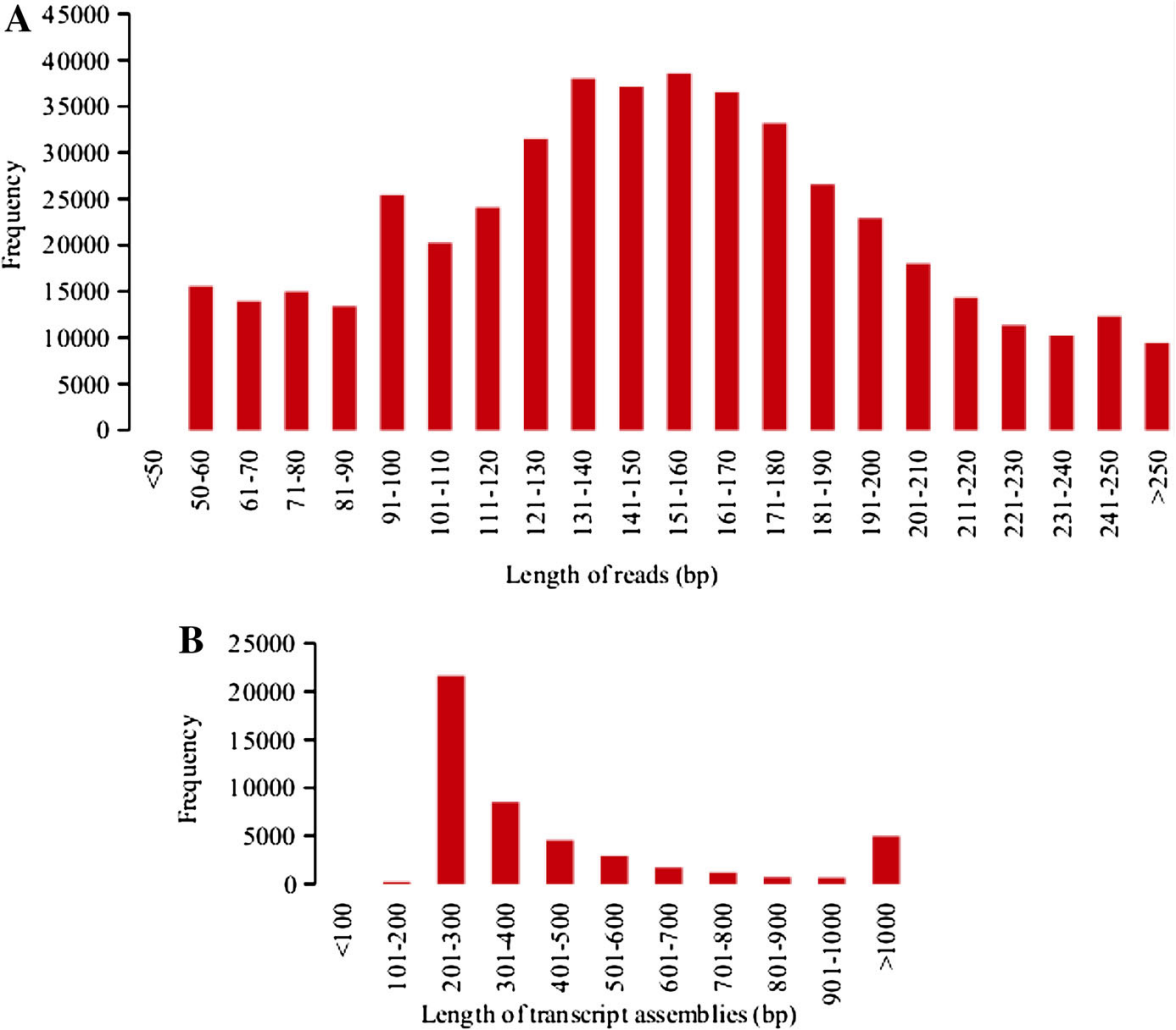
Size distribution of Illumina MiSeq high-quality reads (**a**) and assembled transcripts (**b**). The length of reads and transcript assemblies are given in nucleotide bases

The quality filtered RNA-seq reads were assembled using Velvet and Oases as described in the method section by merging assembled transcripts from all separate assemblies based on *k*-mer values ranging from 19 to 37 with 4 cycles. The resulting contigs merged into 141,506 final transcripts with an N50 of 325 and a mean length of 317 bp was based on the *k*-mer value 23. Out of these, 45,450 that represent contigs clustered into putative loci that denote high confidence value (>0.5) were further subjected to selection for coding regions (Table 2). The size of N50 and mean contig length of each individual assembly varies in the *k*-mer size suggesting the variation in the number of reads incorporated into assemblies at different *k*-mer length (Table 2). The combination of these diverse clusters of contigs resulted in the higher diversity of the representative transcripts in the final merged assembly although fewer transcripts were employed in the merged assembly as a consequence of removing the large sequence reads that were redundant, low quality and too short below the recommended length in the quality trimming process. While the description of assembled contigs and transcripts at different range of *k*-mer size is shown in Table 2, the size distribution of the high-quality reads and the transcript assemblies is indicated in Fig. 2.

**Table 2:** Assembled individual and merged transcripts at different ranges of *k*-mer size

*K*-mer^a^	Assembly		Number of sequences	N50^b^	Mean length (bp)	Total length (bp)
19	Sorted^c^	Contigs	263,614	127	101.3	25,378,706
		Transcripts	22,863	271	257.1	5,877,742
	Singletons^d^	Contigs	178,552	141	105.86	18,901,518
		Transcripts	9724	513	387.68	3,769,817
23	Sorted	Contigs	224,336	148	115.6	25,942,687
		Transcripts	22,574	271	261.0	5,892,229
	Singletons	Contigs	137,865	161	125.6	17,319,022
		Transcripts	9203	497	383.2	3,526,493
27	Sorted	Contigs	203,262	158	129.9	26,399,952
		Transcripts	22,221	265	257.5	5,721,653
	Singletons	Contigs	116,661	172	141.4	16,490,779
		Transcripts	8784	490	380.5	3,342,351
31	Sorted	Contigs	187,573	166	142.2	26,679,412
		Transcripts	21,338	272	265.0	5,654,580
	Singletons	Contigs	100,108	181	156.6	15,677,680
		Transcripts	8767	458	370.8	3,250,911
35	Sorted	Contigs	174,608	174	153.4	26,777,742
		Transcripts	20,050	274	268.8	5,388,755
	Singletons	Contigs	88,112	188	169	14,886,941
		Transcripts	7911	473	381.7	3,019,910
Merged transcripts			141,506	325	317.3	44,893,048
HCV^e^ transcripts			45,450	334	330	15,004,248
LORFs^f^			36,232	516	534.5	13,457,805
Annotated			20,223	534	524	8,231,964

^a^Length-specific subsequence that overlap match between reads

^b^N50 = length-weighted median contig size

^c^Partner paired-end reads

^d^Reads whose partners have been removed

^e^High confidence value (>0.5)

^f^Long open reading frames

### Validation and annotation of transcript assembly

While it is difficult to figure out if a transcript is correctly assembled in a de novo assembly whereby no reference sequence is available, the strategies we employed provided quality transcriptome assembly by identifying and removing the misassembled and retain potentially assembled transcripts. Using an internal validation strategy of mapping quality filtered reads back to the assembly and by identifying ORFs for the potentially assembled transcripts, we removed a total of more than 3220 transcripts that either did not satisfy the minimum per base mean coverage and the minimum length of ORF. In response to filter long ORFs by further screening the 45,450 high confidence transcripts, we identified 36,232 candidate coding transcripts (Table 2).

**Fig. 3:**
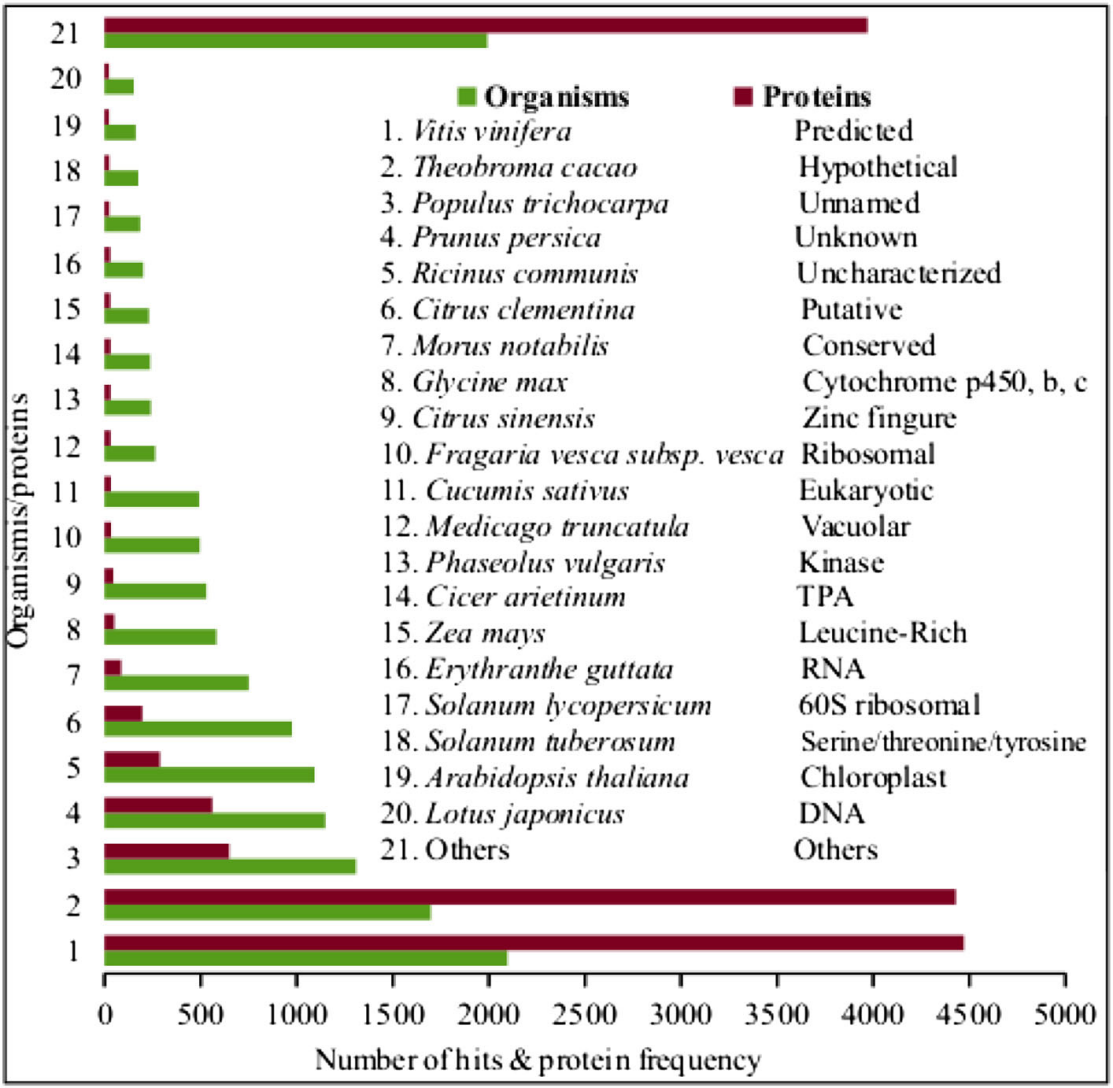
Known proteins queried for homology in the NR protein-database and the corresponding hits. The most related organisms identified with the corresponding number of hits and the frequency of a specific protein type identified in sequence similarity search using BlastP are indicated with *green* and *brown bars*, respectively

**Fig. 4:**
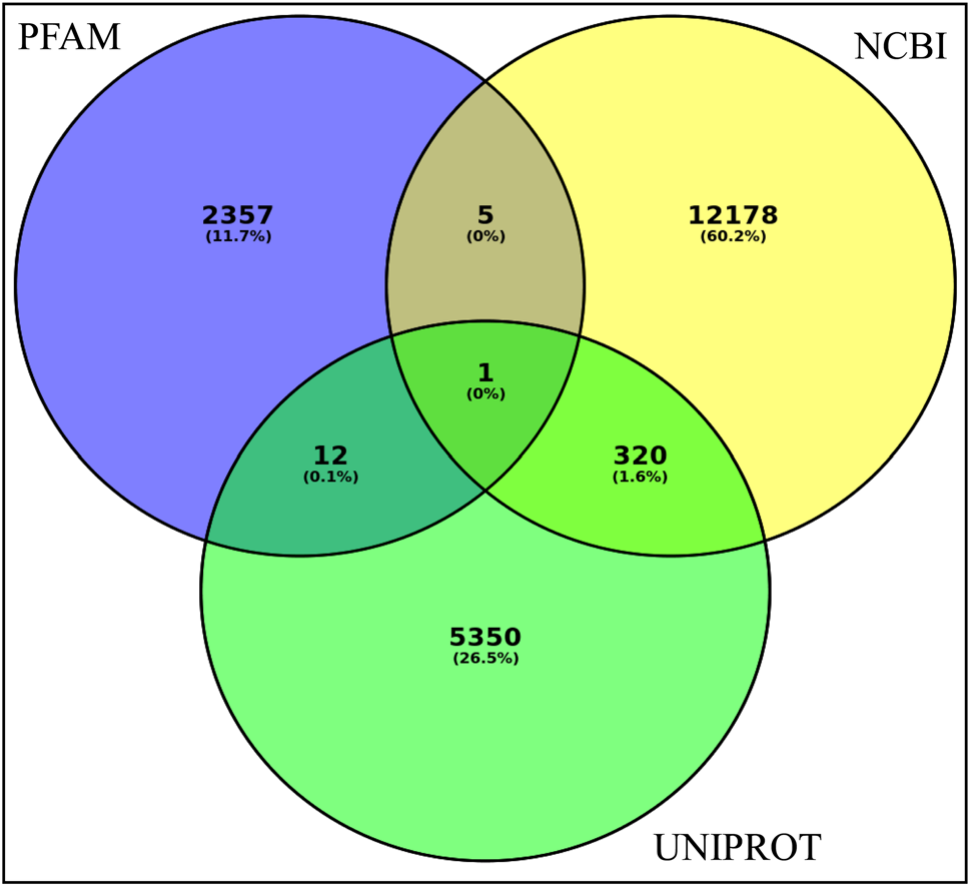
A Venn diagram showing the best hits for protein homologs and conserved domains in the plant protein and PFAM databases. The figure shows the distribution of orthologous genes identified in the three databases. In the overlapping circles, the *numbers* and *percentages* represent the particular genes that were identified in more than one databases in common and in the non-overlapping circles, the protein homologs which are specific to a database are indicated

Blast analysis of these coding transcripts revealed 23,939, 29,671 and 21,800 best hits in the NR NCBI, UNIPROT and PFAM databases, respectively, based on e-value, bit score and sequence identity as described in the “Method” section. This represents mapping of more than 85% coding transcripts to the known protein database accounting for more than 17,800 genes with homology in other species (Table S1; Table S2; Figs. 3, 4) and more than 2300 unique conserved domains (Table 3; Table S3). While 320, 12 and 5 known genes were commonly identified in UNIPROT and NCBI, UNIPROT and PFAM and NCBI and PFAM in pairs, respectively, the 12,178, 5350 and 2357 genes were exclusively identified based on NCBI, UNIPROT and PFAM databases, respectively (Fig. 4). Uricase was identified as a single known gene that was found in all the three databases in common with the respective ids (O04420, UNIPROT; EMS57616.1, NCBI and PF01014.14, PFAM) (Fig. 4). About 20% of the candidate coding transcripts were unmapped to the NR databases which may suggest the uniqueness of the coding regions of the *M. burkeana* leaf de novo transcripts. Of the identified conserved protein domain, Pkinase is the most frequent domain to which 2% of the transcript sequences were mapped (Fig. 3).

**Table 3:** Major classes of conserved protein domains and the number of best hits queried based on PFAM database

Conserved domain type	Clan	No_clan	Total
Coiled-coil	0	2	2
Domain	7917	2913	10,819
Family	5010	4893	9884
Motif	85	20	105
Repeat	950	44	994
Total	13,962	7872	21,834

Predicted, hypothetical, unnamed or unknown, uncharacterized and putative genes together accounted for 70% of the identified proteins in this study substantially supporting the above suggestion that most sequences were uniquely identified. Most protein orthologs were identified from *Vitis vinifera* followed by *Theobroma cacao* and *Populus trichocarpa* (Fig. 3).

### Functional sequence annotation and GO analysis

**Fig. 5:**
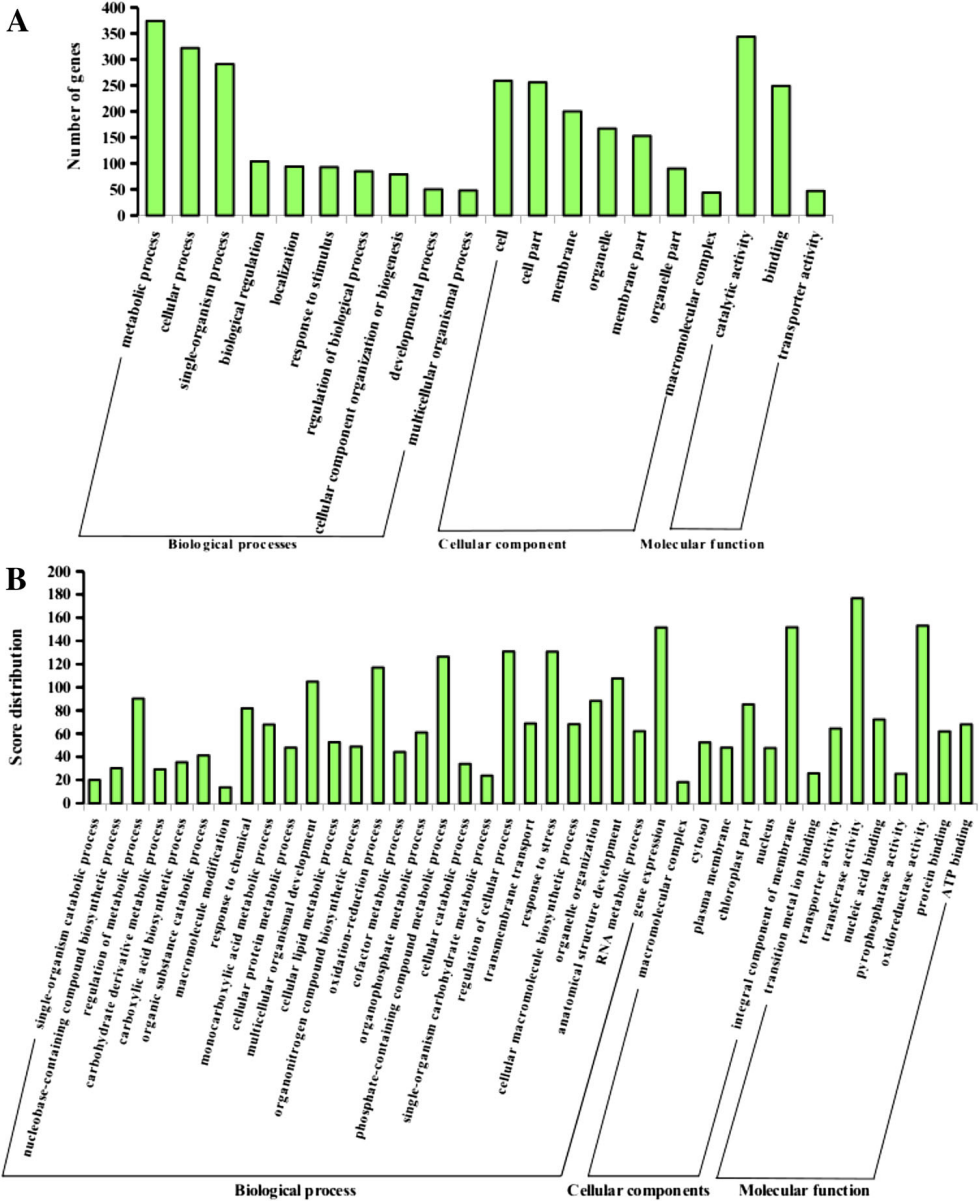
Gene Ontology annotation categorization of the *M. burkeana* leaf transcriptome. **a** Summarized description of the three main GO categories (biological process, cellular component, molecular function) and 20 sub-categories for the GO-terms assigned to the *M. burkeana* coding transcripts based on hits resulted significant number of genes in the NR database. **b** Description of the main GO categories and sub-categories for the GO-terms assigned to *M. burkeana* coding transcripts based on the BLASTX score distribution for mapping genes to the NR database

While all selected sequences submitted to Blast2GO passed through analysis, 31 sequences were blasted to the database without having any significant hit and other 20 sequences were identified with blast hits but without GO and InterProscan annotation, however, no sequence without blast result had InterProscan information. Of the total sequences submitted for annotation, 87.8 and 81.2% received IPS ids and GO-accessions, respectively. On the other hand, while 459 sequences were Blast2GO annotated, 17 sequences had GO mapping without complete annotation such as lacking enzyme commission and pathways mapping. However, of the annotated sequences, at least 80% were mapped to KEGG pathways. The summerized GO terms were shown with the three main GO categories whereby the biological process contributed to 62% of the GO annotations and the cellular component and molecular function each made up to 20% and 18% respectively (Fig. 5).

### Pathway analysis

**Fig. 6:**
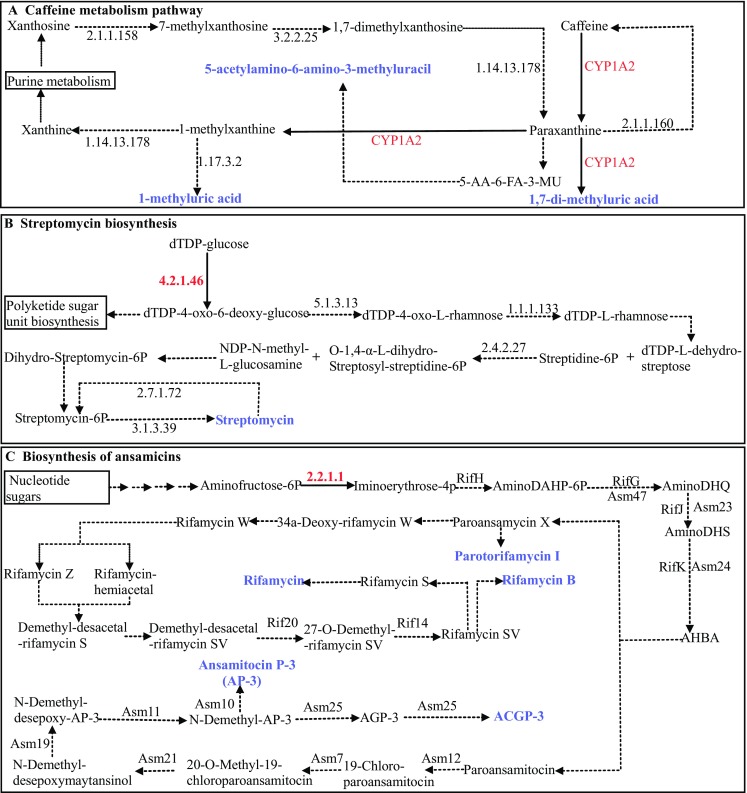
*M. burkeana* leaf coding transcripts involved in the regulation of the caffeine metabolism, biosynthesis of streptomycin and ansamycin pathways. **a** *M. burkeana* leaf coding transcripts involved in the regulation of the caffeine metabolism. **b** *M. burkeana* leaf coding transcripts involved in the regulation of streptomycin biosynthesis pathway. **c** *M. burkeana* leaf coding transcripts involved in the regulation of ansamycin biosynthesis pathway. While caffeine metabolic pathway is specific to tea quality, the biosynthesis of streptomycin and ansamycin pathways are specific to therapeutic medicines. The enzymes encoded by the *M. burkeana* leaf transcripts are indicated by *red enzyme numbers* (EC-number) and all other enzymes are indicated by *black*. The chemical reactions catalyzed by the enzymes encoded by the *M. burkeana* leaf transcripts are indicated in *black solid arrows* and all other reactions and intermediates are indicated in *black dashed*/*dotted arrows*. End products are indicated in *blue color* and *bold*. *5-AA-6-FA-3-MU* 5-acetylamino-6-formylamino-3-methyluracil

In order to identify and functionally classify the metabolic pathways that are biologically active in *M. burkeana* leaf tissues, we performed KEGG pathway analysis using Blast2GO functional annotation platform. Among 36,232 validated transcript coding regions we used the top 500 coding transcripts (CDs) selected by Transdecoder with minimum of 1250 bases long for pathway analysis. A total of 93 KEGG pathways were identified to which 93.4% of the coding transcripts were mapped all annotated with enzymes (EC numbers). The pathways were classified into 15 major functional categories based on the canonical classes of the pathway maps in the KEGG database (Table 4). Among others, the biosynthesis of secondary metabolites involves 8.5% of the pathways including caffeine metabolism, flavonoid, phenylpropanoid and streptomycin biosynthesis just to name but a few which are associated to 5.4% of the candidate coding transcripts (Fig. 6; Table S4).

**Table 4:** Functional categories of the metabolic pathways identified and involved in the tea quality and medicinal values

Major functional categories	Pathways involved (number, %)	% of sequences assigned
Amino acid metabolism	15, 16	19.5
Carbohydrate metabolism	14, 15	23.3
Lipid metabolism	14, 15	10.7
Xenobiotics biodegradation and metabolism	10, 10.6	3.9
Metabolism of terpenoids and polyketides	9, 9.6	13.7
Biosynthesis of other secondary metabolites	8, 8.5	5.4
Energy metabolism	5, 5.3	8.4
Metabolism of cofactors and vitamins	5, 5.3	3.4
Metabolism of other amino acids	5, 5.3	3.9
Glycan biosynthesis and metabolism	3, 3.2	1.7
Nucleotide metabolism	2, 2.1	3.4
Carbon metabolism	1, 1.1	1.7
Environmental information processing; signal transduction	0.2	0.2
Genetic information processing; translation	1, 1.1	0.6
Organismal systems; immune system	1, 1.1	0.2

### Metabolic evidence for medicinal and tea beverage property of *M. burkeana*

Metabolic pathways analysis in the current study shows ample evidence that *M. burkeana* is a potential plant with different sources of naturally occurring chemicals associated with various antibiotic and tea beverage features. The three peculiar categories of secondary metabolic pathways discovered with medicinal and tea purposes in *M. burkean*a leaf transcriptome are: (1) xenobiotics biodegradation and metabolism; (2) metabolism of terpenoids and polyketides and (3) biosynthesis of other secondary metabolites (Table 4; Table S4). It is worth describing these major categories in further detail in relation to the medicinal and tea quality impact they may have in *M. burkeana* leaf tissue.

#### Xenobiotics biodegradation and metabolism

The xenobiotics category of the metabolic pathways consists of 10 metabolic pathways to which 4% of the de novo assembled coding transcripts in leaf tissue were attributed. Among others, chloroalkane and chloroalkene degradation [PATH:ko00625] frequently mapped the transcript sequences. The pathways are aminobenzoate degradation [PATH:ko00627], benzoate degradation [PATH:ko00362], caprolactam degradation [PATH:ko00930], drug metabolism—cytochrome p450 [PATH:ko00982], drug metabolism—other enzymes [PATH:ko00983], ethylbenzene degradation [PATH:ko00642], metabolism of xenobiotics by cytochrome p450 [PATH:ko00980], styrene degradation [PATH:ko00643] and toluene degradation [PATH:ko00623]. Since xenobiotics are synthetic chemicals that are refractory to degradation, understanding of the molecular basis of these metabolic pathways will provide successful means for biological application of their biodegradability. This suggests that *M. burkeana* leaf transcriptome encodes genes with necessary molecules for biosafety of environmental xenobiotics.

#### Metabolism of terpenoids and polyketides

This category consists of 9 different metabolic pathways associated with 13.8% of the candidate coding transcripts. These include biosynthesis of ansamycins [ko01051] (Fig. 6), biosynthesis of vancomycin group antibiotics [PATH:ko01055], carotenoid biosynthesis [PATH:ko00906], geraniol degradation [PATH:ko00281], insect hormone biosynthesis [PATH:ko00981], limonene and pinene degradation [PATH:ko00903], polyketide sugar unit biosynthesis [PATH:ko00523] and terpenoid backbone biosysnthesis [PATH:ko00900]. The most frequent metabolic pathway in this category with 68.7% of the transcript sequences assigned to this group was antibiotic biosynthesis. Better understanding of these metabolic pathways gives insight into medicinal application of terpenoids and polyketides to which *M. burkeana* leaf transcriptome encoded genes are attributed.

#### Biosynthesis of other secondary metabolites

The *M. burkeana* leaf tissue originated coding transcripts were identified to be involved in eight metabolic pathways in this category (Table 4; Table S4). Previously, three secondary metabolic pathways of tea-specific compounds were identified using the *Camellia sinensis* transcriptome (Shi et al. 2011) among which caffeine and flavonoid are common with what we currently identified for *M. burkeana* leaf transcriptome. However, in the current investigation, we identified additional metabolic pathways involved in the biosynthesis of secondary metabolites such as Flavone and Flavonol biosynthesis ([PATH:ko00944] and Phenylpropanoid biosynthesis [PATH:ko00940] with specificity to tea beverage and medicinal aspects (Ververidis et al. 2007) and Isoquinoline alkaloid biosynthesis [PATH:ko00950] Novobiocin biosynthesis [PATH:ko00401], Streptomycin biosynthesis [PATH:ko00521] and Tropane, piperidine and pyridine alkaloid and biosynthesis [PATH:ko00960] with more of antibiotic and pharmacological properties (Cushnie et al. 2014). This suggests that our work adds new data to the existing database indicating that *M. burkeana* leaf tissue is a potent reservoir of medicinally indispensable natural products involved in various biological activities such as stimulant effect, defense and immunity system, and antibiotic features.

In caffeine metabolism [PATH:ko00232] (Fig. 6), Cytochrome P450 1A2 (CYP1A2), a monooxygenase [EC:1.14.14.1], catalyzes the conversion of caffeine to paraxanthine and then to 1,7-dimethyluric acid and to 1-methylxanthine. This major gene is a rate limiting in the reaction as it begins and partly ends the conversion of caffeine. As the two largest groups of naturally occurring flavonoids in plants, flavones and flavonols [PATH:ko00944] were identified in this study (Table S4) which have been identified to have a significant impact in the survival of the plant by re-constituting the redox regulation of proteins, transcription factors and signal transduction thereby involved in anti-oxidation and neuron protection (Dajas et al. 2013). The proper functioning of the green tea is specifically effected by flavanols and flavonols which account for 30% of fresh leaf dry weight (McKay and Blumberg 2002). The phenylpropanoid pathway [PATH:ko00940] to which 24% of the leaf coding transcripts were assigned, is also extremely important metabolic pathway in this category. The indispensability of this pathway is not just that it is a base for the synthesis of important compounds like the flavonoids, coumarins, and lignans, but because of its place in hydroxycinnamyl alcohols (monolignols) production (Boerjan et al. 2003).

#### Pathways associated with primary products

Sixty-five KEGG pathways involved in the biosynthesis of primary products were identified which accounts for 69.9% of the total metabolic pathways. About 77% of the coding transcripts were assigned to the primary pathways involved in the biosynthesis of chemical products essential for plant survival. A considerable number of coding transcripts were identified to have mapped to multiple pathways up to 20 KEGG pathways (Table S4). This suggests that *M. burkeana* leaf transcriptome encode for genes regulating multiple enzymatic functions most of which associated with plant life and survival.

### Gene enrichment network

**Fig. 7:**
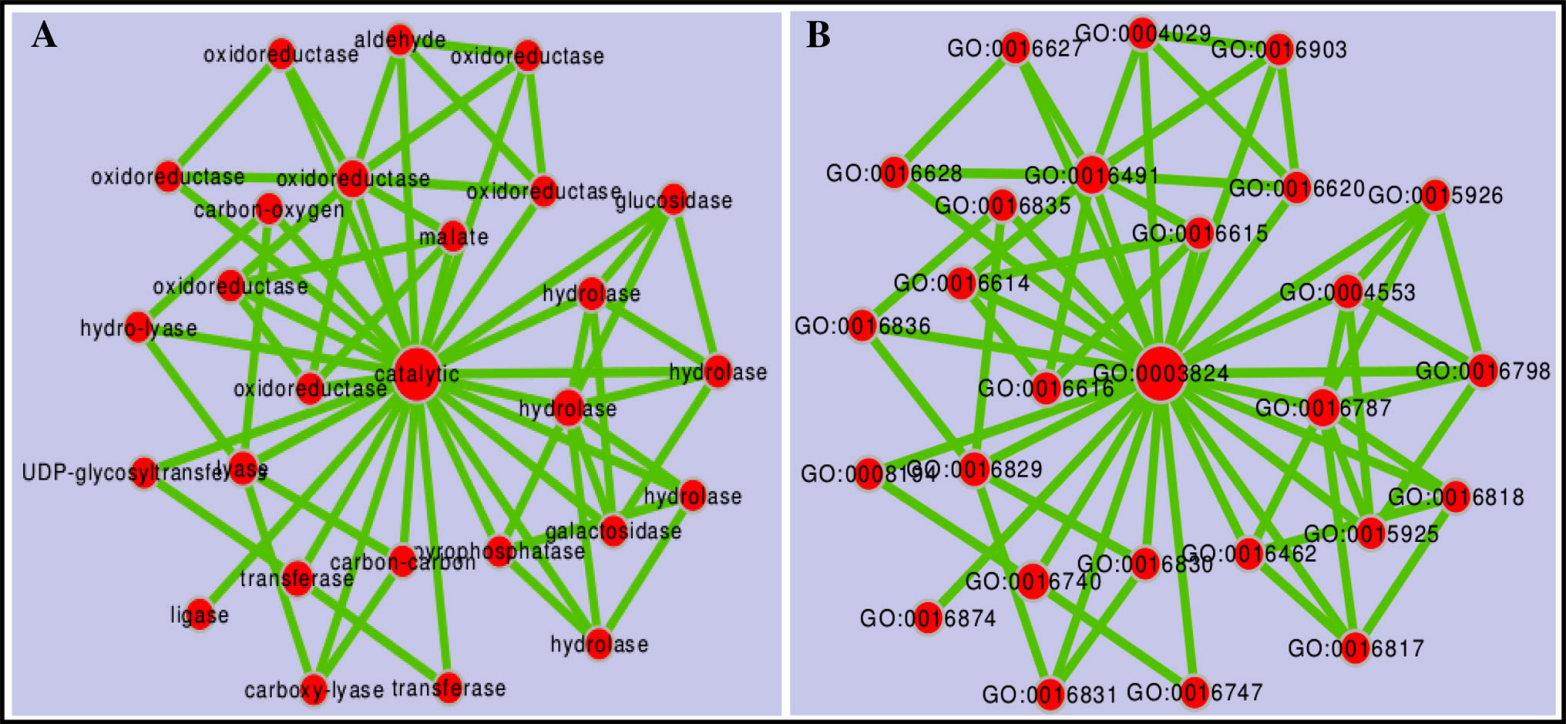
Gene enrichment network map based on the molecular function of the Gene Ontology category. **a** Gene enrichment network map for a significantly enriched gene members in the gene families that encode enzymes for the key pathways associated with molecular function. **b** Gene enrichment network map for the GO-accessions that correspond to the members of the gene families to show the correlation of the GO-terms assigned to the coding transcripts of the *M. burkeana* leaf tissue with the significantly enriched genes encoded by the same coding transcripts and involved in the key metabolic pathways. The gene enrichment network map was produced using Cytoscape v 3.3.0

Gene enrichment network map was constructed selectively for 35 genes associated with enriched GO-terms using Cytoscape (Shannon et al. 2003). Of the total submitted GO-accessions to AgriGO (Du et al. 2010) 80 GO-terms for BP, 27 for CC and 35 for MF were find to be enriched. We used the GO-terms enriched for the MF to establish the gene enrichment network map by linking with the gene families that encode for catalytic enzymes. Figure 7 shows interactive biological networks based on the coding transcript sequences enriched for GO-terms related to MF of the GO categories.

### Phylogeny

**Fig. 8:**
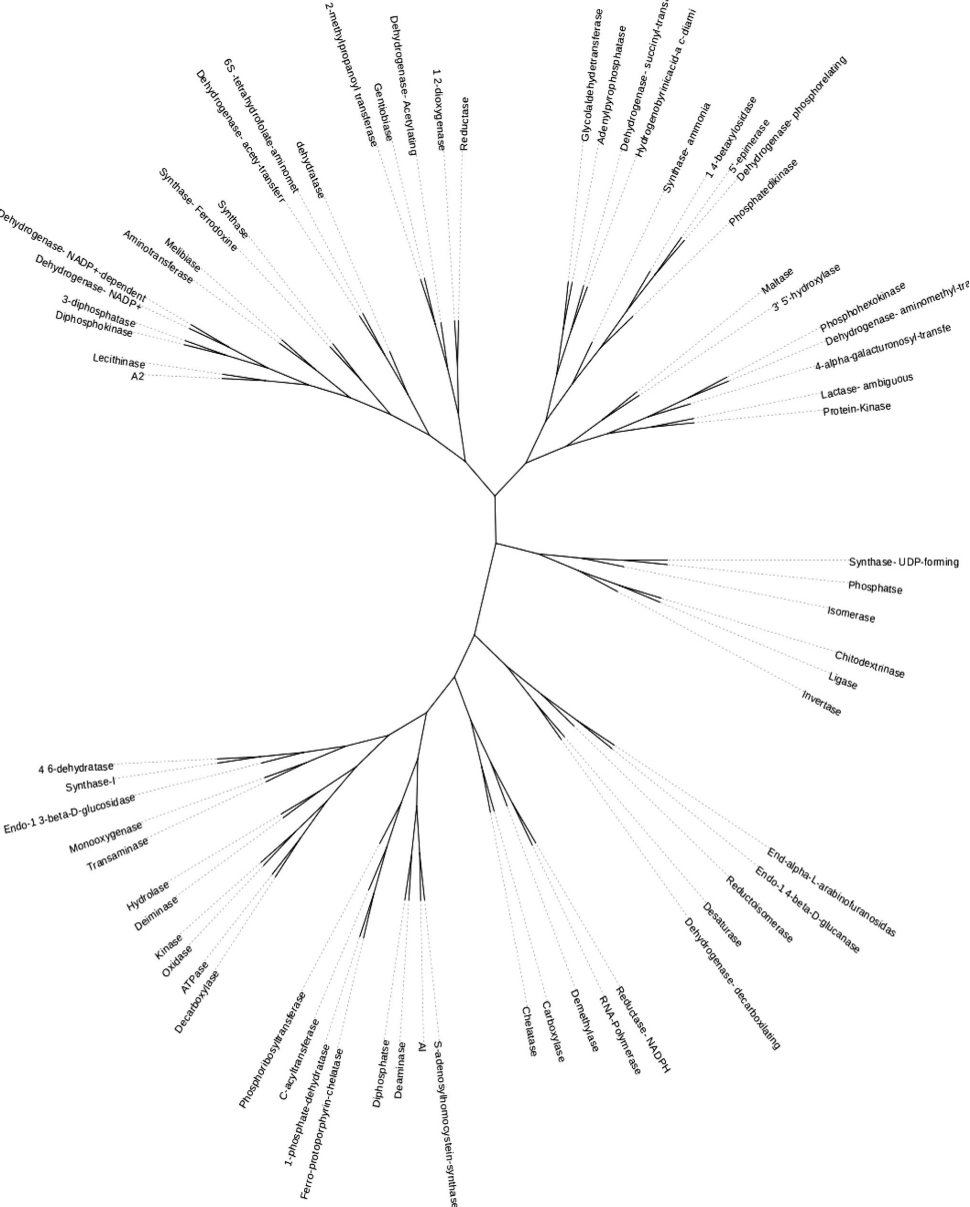
Unrooted phylogenetic tree of *M. burkeana* leaf transcriptome encoded gene families involved in the metabolic pathways. This phylogenetic tree shows the relatedness of the gene families that encode the key enzymes involved in the major metabolic pathways identified in the study. The tree was generated by ClustalW2 phylogeny (http://www.ebi.ac.uk/Tools/phylogeny/clustalw2_phylogeny/) and visualized using interactive Tree of Life (iTOL), an open source program

The gene families involved in the biochemical activities associated with the pathways for the primary and secondary products in *M. burkeana* leaf tissue were classified in 15 major groups congruent with the number of major functional classes of the metabolic pathways identified in this study. The distinction of the groups may be attributed to their evolutionary and functional character. Unrooted phylogenetic tree was used to show the relatedness of the gene families (Fig. 8).

## Discussion

Plant secondary metabolites and their derivatives have evolved to become an extremely useful source of therapeutic agents (Koehn and Carter 2005). In this era of drug discovery, the methods for unraveling biosynthetic potential of these novel products have shifted from ordinary analysis of antibacterial and antifungal activity to genetic transformation based assays of eukaryotic cells with efficient regulation of gene expression and inhibition and detection of small-molecule-receptor interaction (Gullo 1994). Molecular biology techniques combined with the advent of next generation sequencing and bioinformatic tools has provided dependable strategy to synthesize and screen these natural life-saving products particularly from non-model plants. A herbal crop *M. burkeana,* used for tea flavor and traditional medicine, is one of these plants with phytotherapeutic potential. Despite this, to date, little has been studied about this crop and no genomic and transcriptomic information is available in literature. Whole genome and transcriptome sequencing have been commonly used in many studies (Shi et al. 2011; Miller et al. 2012; Gupta et al. 2013) using both Illumina and 454 pyrosequencing platforms to investigate the natural products associated with tea and pharmaceutical properties in several non-model plants. In this study, we used the Illumina MiSeq platform to generate RNA-seq reads to establish a partial transcriptome profiling from leaf tissue in *M. burkeana* and to reconstruct and functionally annotate de novo transcript assembly to explore key metabolic pathways and associated putative genes that determine the biosynthesis of novel secondary metabolites with tea and medicinal values.

The Illumina MiSeq RNA sequencing platform has generated 2,590,652 paired-end reads that were assembled using *k*-mer values ranging from 19 to 37 with 4 cycles into 45,450 high-quality transcripts selected out of the final merged transcripts and 36,232 long ORFs with an N50 of 516 and a mean length of 535 bps. These were validated and annotated using NR protein databases to identify putative genes for key metabolic pathways involved in biosynthesis of secondary metabolites from *M. burkeana.* This data indicates the basic knowledge particularly in terms of harvestable potential products harbored in leaf tissue of the plant.

Several other works have investigated transcriptome profiling on a large scale on tea plants (Shi et al. 2011; Miller et al. 2012; Wang et al. 2013) in the presence of at least some datasets from related species. Despite the availability of limited resources on this species, in order to generate improved and efficient transcriptome dataset from leaf tissue, the best strategy was employed to include RNA fragment-based construction of Illumina library with the aim to enhance uniformity and to decrease RNA secondary structure (Mortazavi et al. 2008). The sequencing depth and efficiency of de novo assembly was improved through a paired-end sequencing library and through applying a strategy for reads quality insurance (Zhang et al. 2011; Guo et al. 2013). Multiple *k*-mer values with several cycles were used to maximize the assembly of each transcriptome (Zerbino and Birney 2008; Schulz et al. 2012). The use of validated long open reading frames also provided reliable option for gene annotation in three NR public databases so as to gain more or less complete biological and functional information.

Based on these strategies, the best hits queried against the NR NCBI, UNIPROT and PFAM databases accounted for 61.5, 76.2 and 56% of the total validated candidate coding transcripts used, respectively. This was linked not only to a total of more than 17,800 known genes with homology from other species, but also to more than 2300 conserved domains. Approximately 20% candidate coding transcripts did not provide any significant mapping with the known genes in the NR databases. While various reasons could be suggested to the cause of this, the uniqueness of the coding regions of our de novo transcript assembly in the leaf tissue of *M. burkeana* may be one of the possible factors. The top 500 long coding transcripts among the total transcripts that provided the best hits were selected to assign the functional GO annotation and to map the KEGG pathways. The GO annotation with all the three GO categories namely biological process, cellular components and molecular function showed a considerable number of GO-term assignments to the coding transcripts, suggesting a richness of the *M. burkeana* leaf transcriptome data in diverse source of genes and gene products. Mapping the coding transcripts onto KEGG pathways enabled to discover particular coding transcripts with the potential to map up to 20 KEGG pathways that are involved in biochemical synthesis of secondary metabolites in every pathway they map. Annotation of such transcripts with enzyme commission reveals a diverse source of genes encoding for most of the enzymes involved in the biochemical synthesis of secondary metabolites.

By and large, a total of 93 KEGG pathways were identified to which 93.4% of the transcript coding regions were mapped which are all annotated with enzymes numbers. The 15 major categories of the KEGG pathways to which these coding transcripts were classified, shows the functional diversity of these transcripts in coding for major genes for key metabolic pathways involved in the biosynthesis of secondary metabolites specific to tea quality and medicinal value. In particular, secondary metabolic pathways represent 30.1% of all the pathways identified in association with tea and therapeutic medicine. These, in combination with the other 65 pathways involved in the biochemical synthesis of primary products and a total of more than 80 gene families encoding for the key enzymes involved in a various biochemical reactions were considered viable evidence that *M. burkeana* leaf transcriptome provides a rich source of information in particular association with tea quality and medicinal purpose.

While comprehensive dataset is important for detailed functional information and overall transcriptome profiling in *M. burkeana*, the results obtained in the current study provided an insight into secondary metabolites involved in therapeutic medicine and tea fragrance revealing a considerable number of putative genes for key metabolic pathways associated with these products suggesting the quality of our de novo transcript dataset in providing biologically relevant information. Thus, *M. burkeana* denotes a classical example of the herbal plants that demonstrate medicinal and tea quality and can contribute to the needs of the broader community medicinally and otherwise. This data provides new knowledge which would be of great benefit to the public and the medical industry as well as in further comparative studies.

## Electronic supplementary material

Below is the link to the electronic supplementary material.

Supplementary material 1 (DOC 34 kb)

Table S1. Description of functional protein orthologs identified based on NCBI database (XLSX 23 kb)

Table S2. Description of selected functional orthologs identified based on UNIPROT database (XLSX 22 kb)

Table S3. List of the best unique conserved protein domains identified based on PFAM database (XLSX 20 kb)

Table S4. Description of the KEGG pathways and functional categories identified for *M. burkeana* leaf tissue (XLSX 20 kb)

## Abbreviations

BLAST: Basic local alignment search tool
BP: Biological processes
CC: Cellular components
GO: Gene ontology
iTOL: Interactive tree of life
KEGG: Kyoto Encyclopedia of Genes and Genomes
MF: Molecular functions
NCBI: National Center for Biotechnology Information
NGS: Next generation sequencing
NR: Non-redundant
ORFs: Open reading frames
QC: Read quality control
