# Supplementary material for: De novo transcriptome sequencing in Monsonia burkeana revealed putative genes for key metabolic pathways involved in tea quality and medicinal value
Source: 3 Biotech. 2016 Nov 19;6(2):250. doi: 10.1007/s13205-016-0563-y (PMC5116299; doi:10.1007/s13205-016-0563-y)
Supplement: Supplementary file 1 — Supplementary material 1 (DOC 34 kb) [file 13205_2016_563_MOESM1_ESM.doc]

Supplementary Fig. 1. The first 20 most frequent conserved protein domains in the PFAM database**.**

1. Vitis vinifera Predicted

Vitis vinifera Predicted

Theobroma cacao Hypothetical

Populus trichocarpa Unnamed

Prunus persica Unknown

Ricinus communis Uncharacterized

Citrus clementina Putative

Morus notabilis Conserved

Glycine max Cytochrome

Citrus sinensis Zinc

Fragaria vesca subsp. vesca Ribosomal

Cucumis sativus Eukaryotic

Medicago truncatula Vacuolar

Phaseolus vulgaris Kinase

Cicer arietinum Tpa

Zea mays Leucine-Rich

Erythranthe guttata Rna

Solanum lycopersicum 60S

Solanum tuberosum Serine

Arabidopsis thaliana Chloroplast

Lotus japonicus Dna

Others Others

1. Theobroma cacao Hypothetical
2. Populus trichocarpa Unnamed
3. Prunus persica Unknown
4. Ricinus communis Uncharacterized
5. Citrus clementina Putative
6. Morus notabilis Conserved
7. Glycine max Cytochrome
8. Citrus sinensis Zinc
9. Fragaria vesca subsp. vesca Ribosomal
10. Cucumis sativus Eukaryotic
11. Medicago truncatula Vacuolar
12. Phaseolus vulgaris Kinase
13. Cicer arietinum Tpa
14. Zea mays Leucine-Rich
15. Erythranthe guttata Rna
16. Solanum lycopersicum 60S
17. Solanum tuberosum Serine
18. Arabidopsis thaliana Chloroplast
19. Lotus japonicus Dna
20. Others Others
21. Vitis vinifera
22. Theobroma cacao
23. Populus trichocarpa
24. Prunus persica
25. Ricinus communis
26. Citrus clementina
27. Morus notabilis
28. Glycine max
29. Citrus sinensis
30. Fragaria vesca subsp. vesca
31. Cucumis sativus
32. Medicago truncatula
33. Phaseolus vulgaris
34. Cicer arietinum
35. Zea mays
36. Erythranthe guttata
37. Solanum lycopersicum
38. Solanum tuberosum
39. Arabidopsis thaliana
40. Lotus japonicus
41. Others
